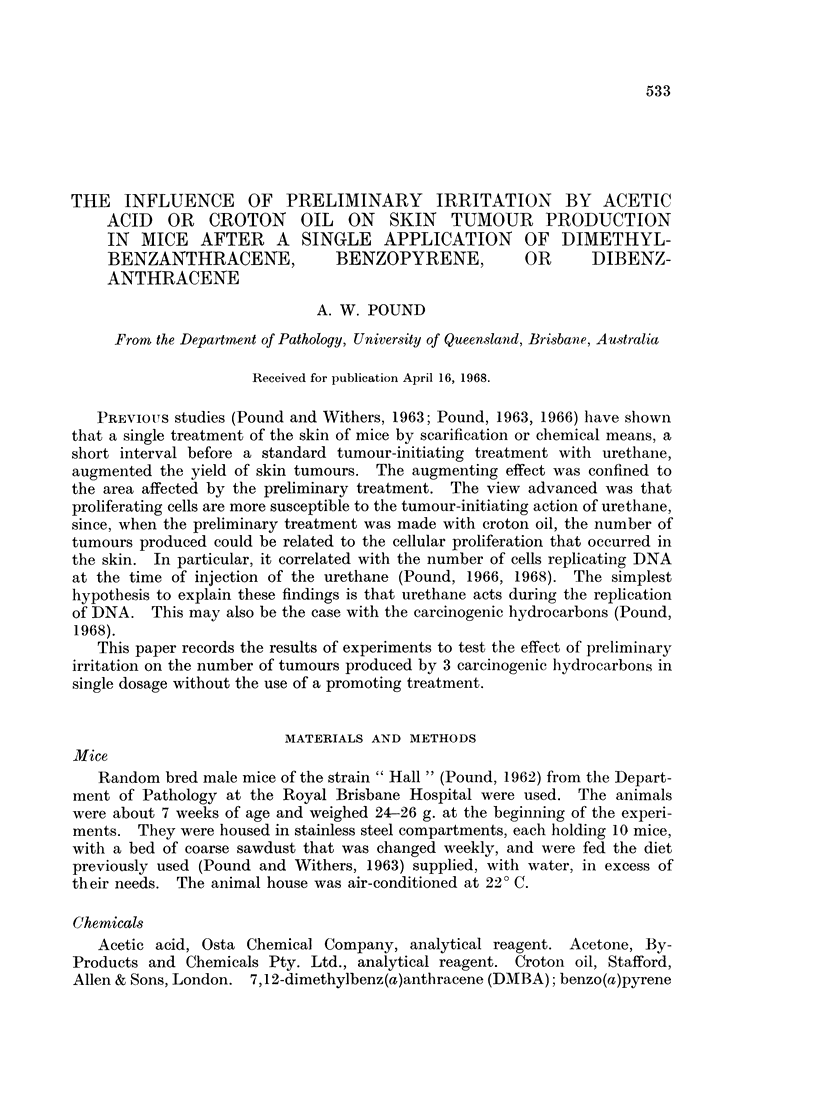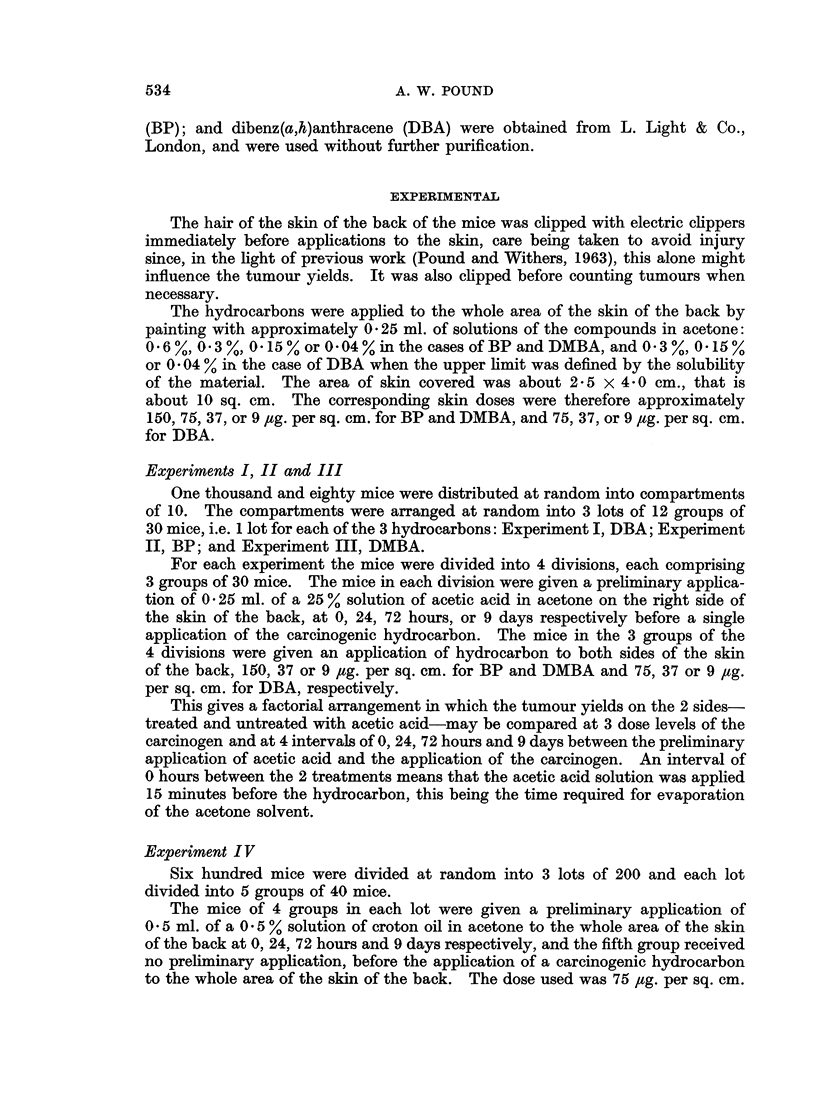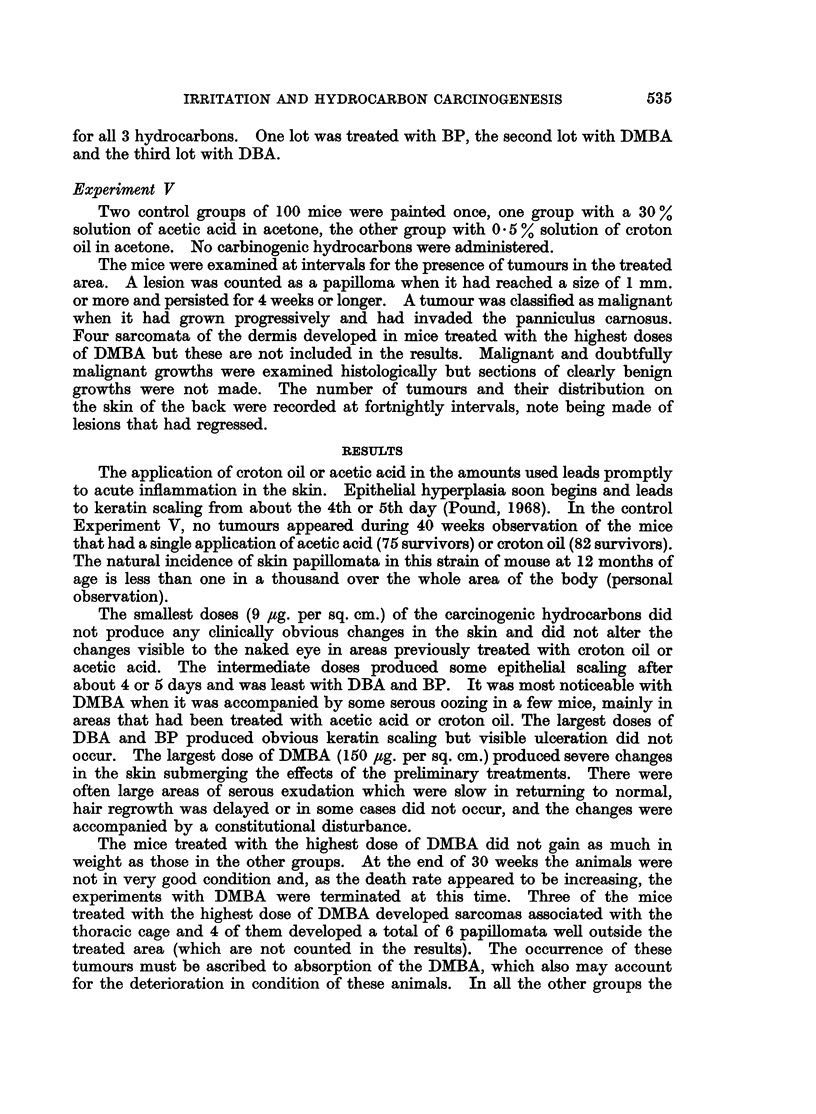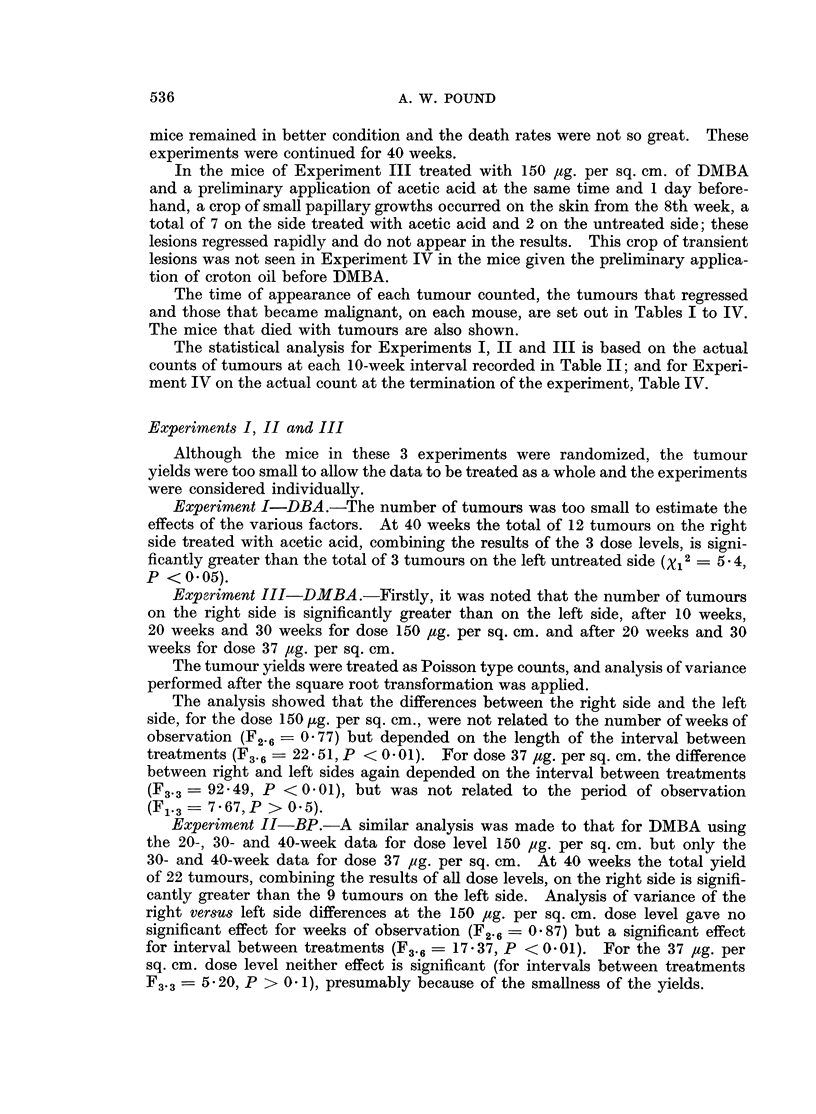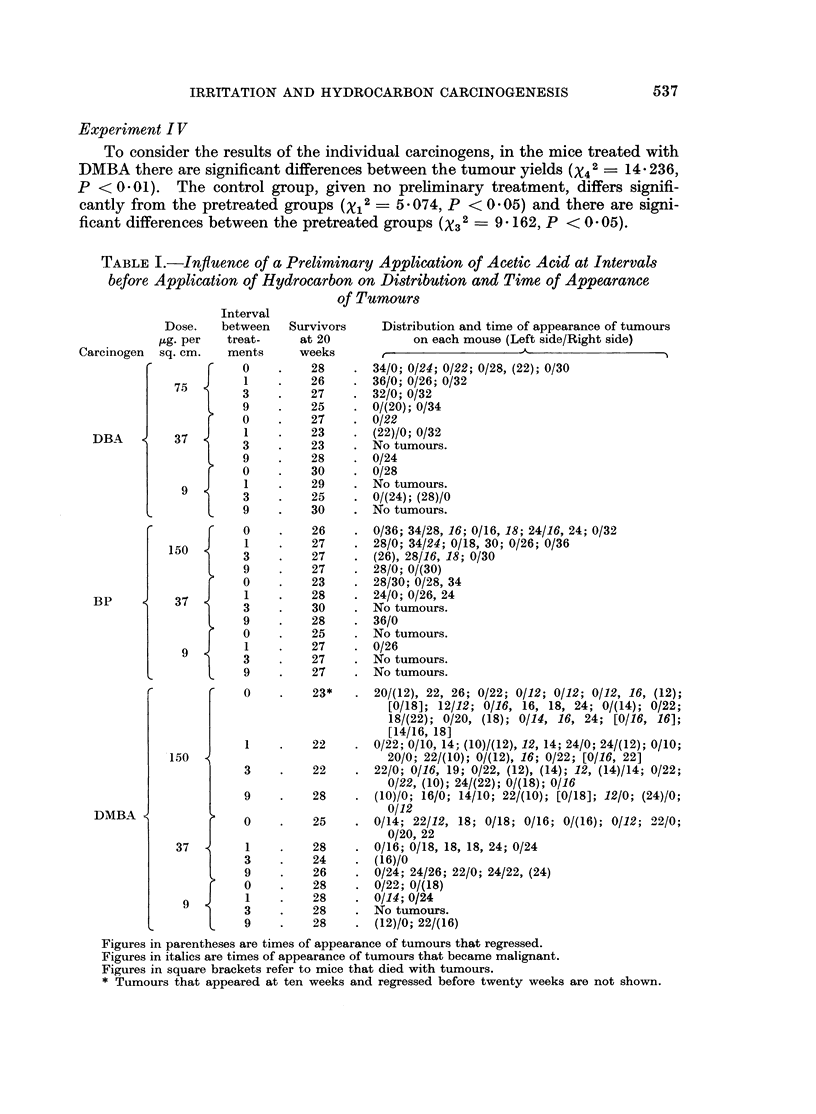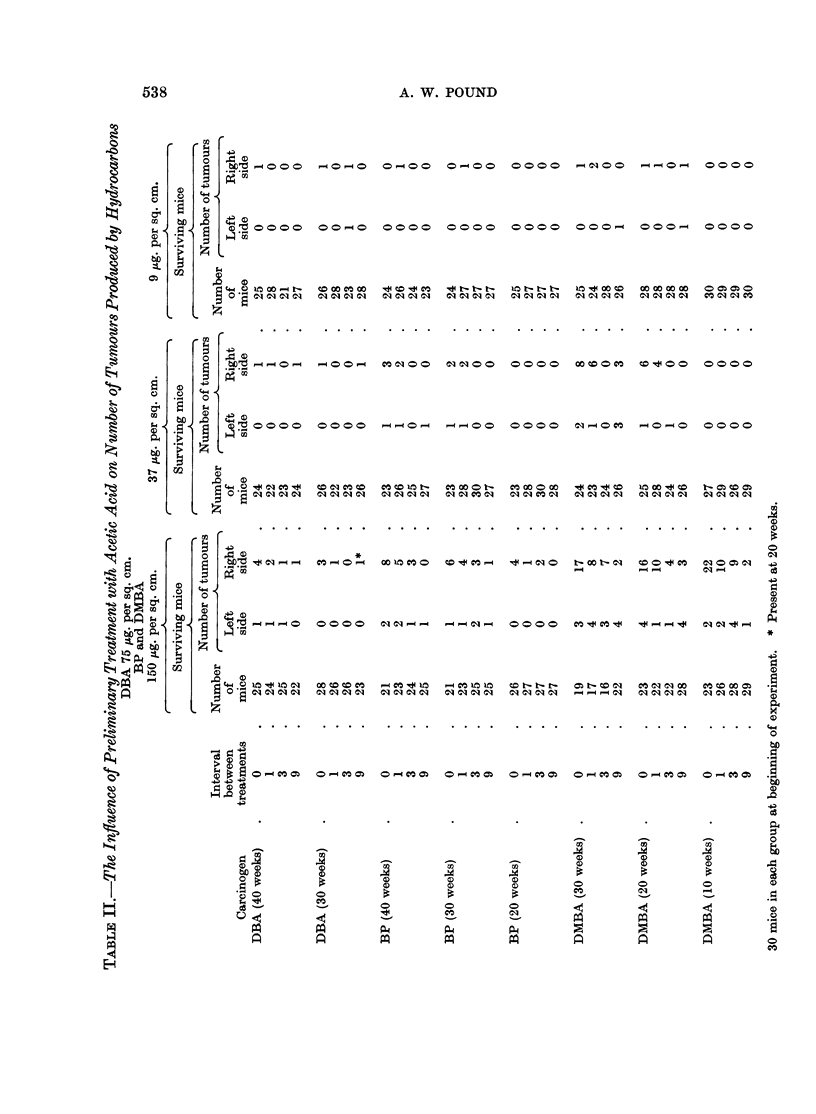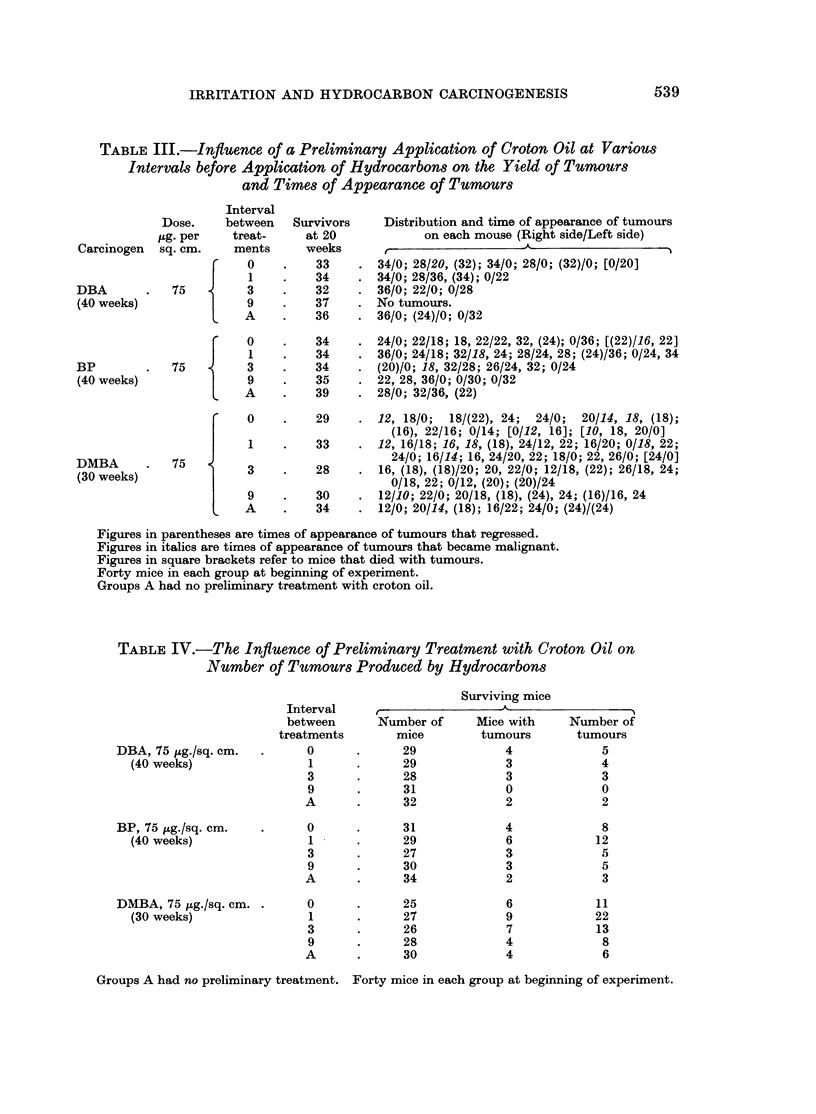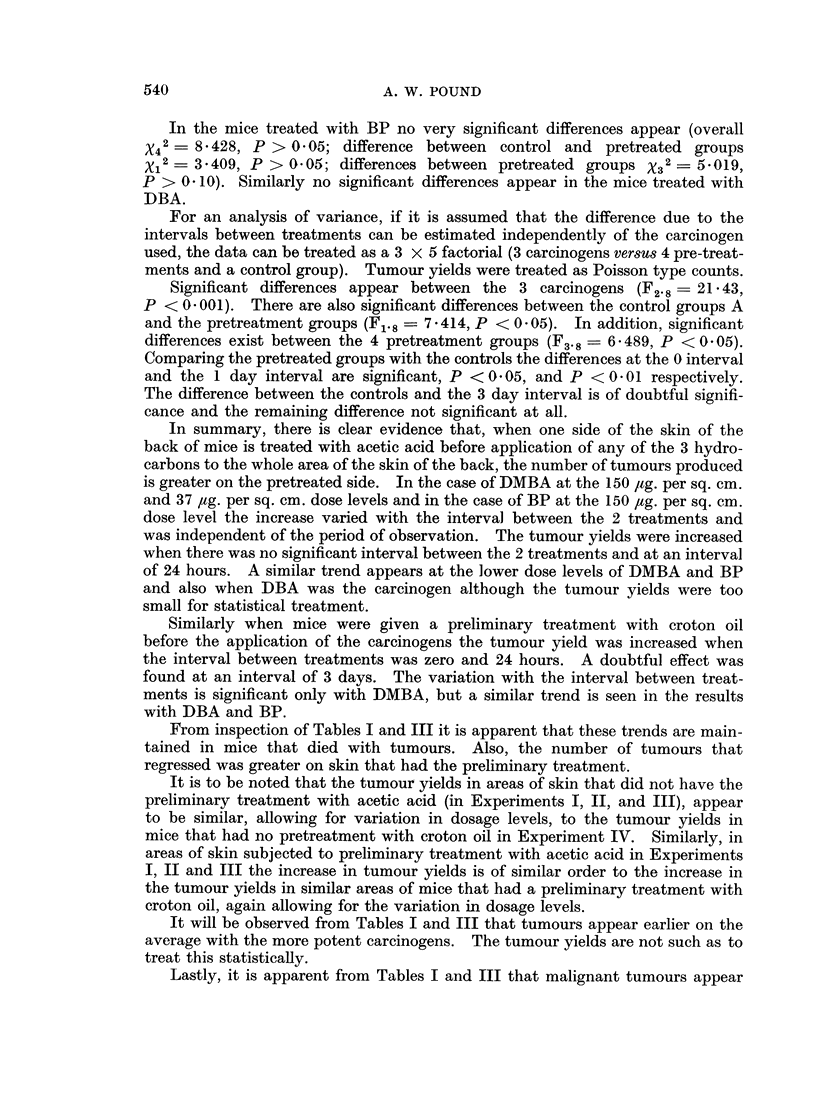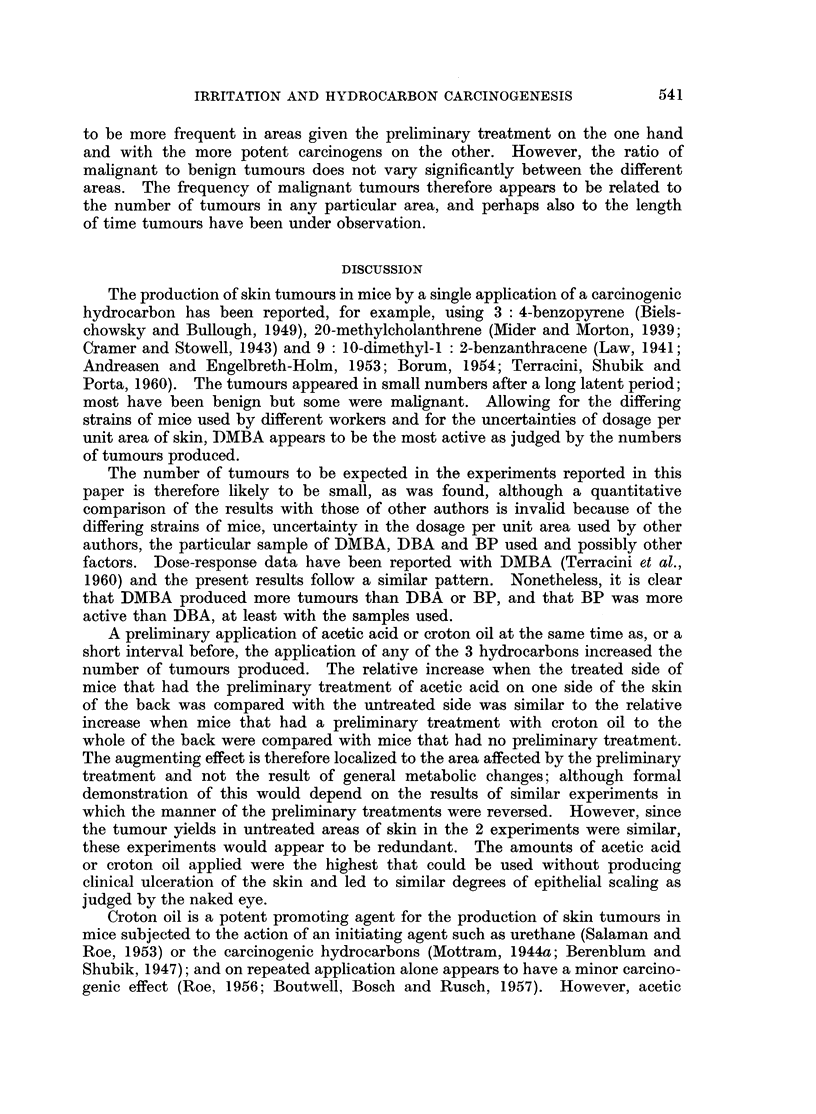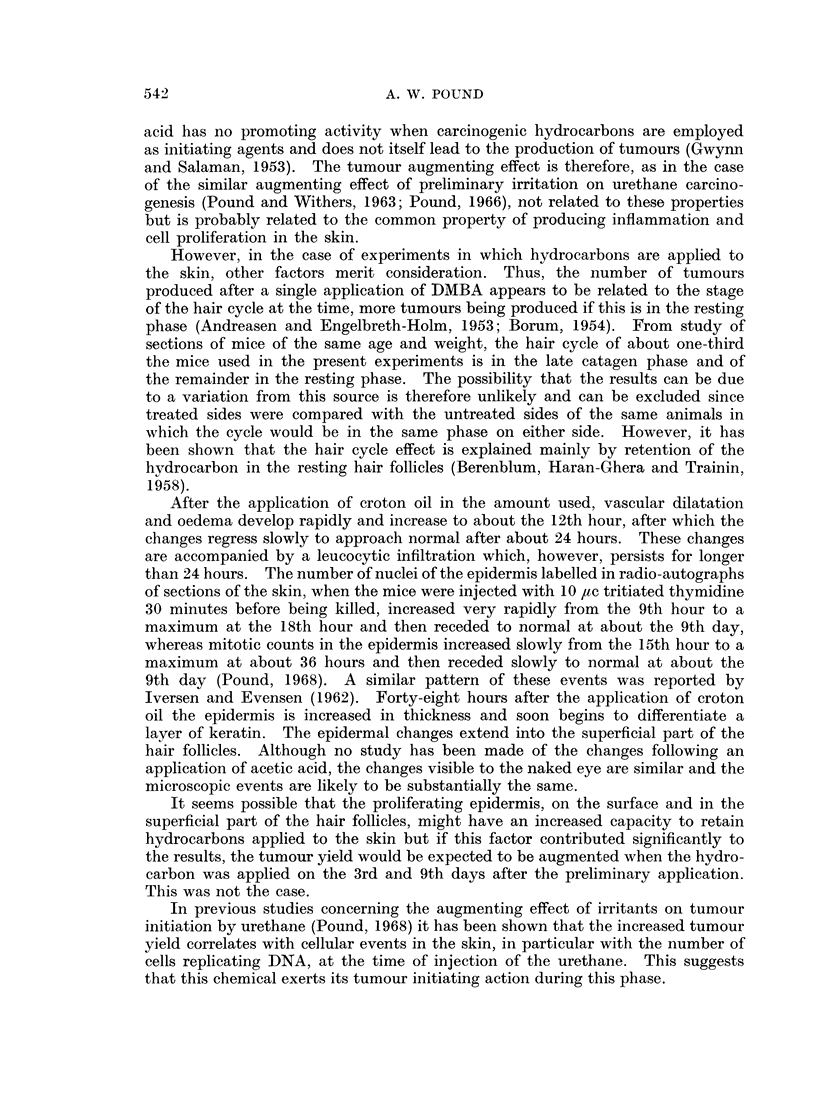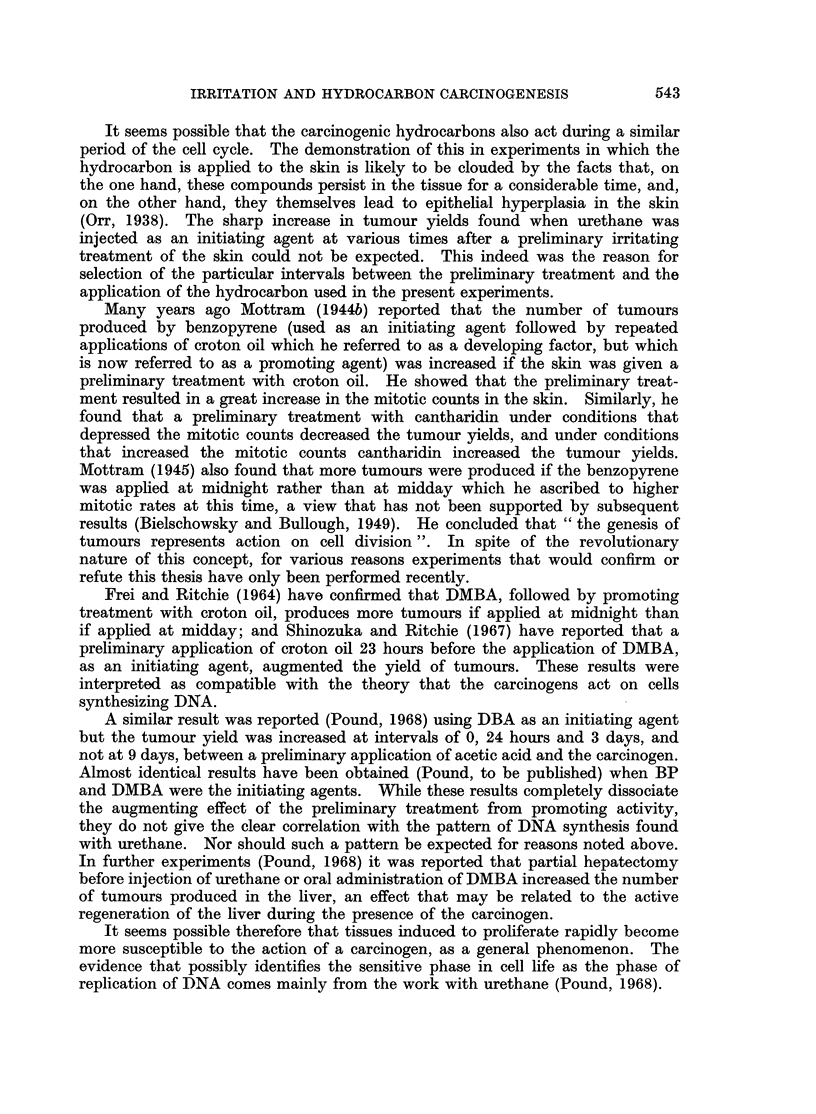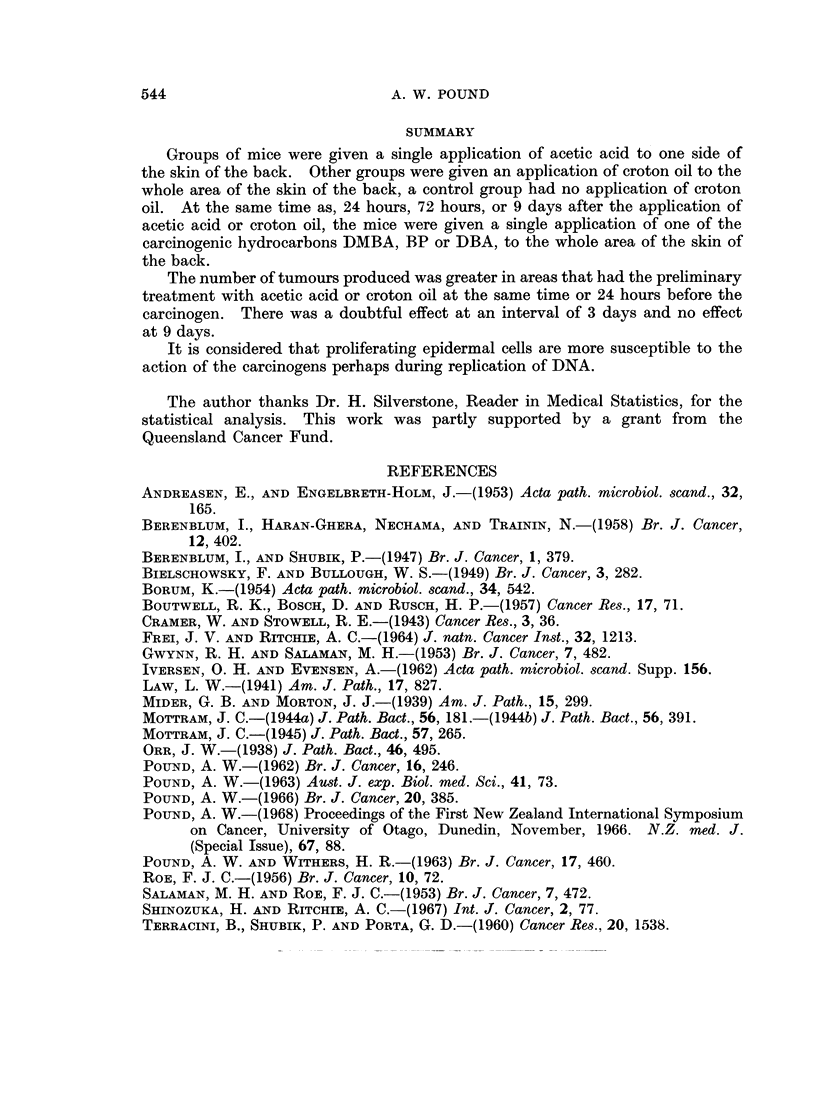# The influence of preliminary irritation by acetic acid or croton oil on skin tumour production in mice after a single application of dimethyl-benzathracene, benzopyrene, or dibenzanthracene.

**DOI:** 10.1038/bjc.1968.64

**Published:** 1968-09

**Authors:** A. W. Pound


### THE INFLUENCE OF PRELIMINARY IRRITATION BY ACETIC ACID OR CROTON OIL ON SKIN TUMOUR PRODUCTION IN MICE AFTER A SINGLE APPLICATION OF DIMETHYLBENZANTHRACENE, BENZOPYRENE, OR DIBENZANTHRACENE

A. W. POUND

*From the Department of Pathology, University of Queensland, Brisbane, Australia*

Received for publication April 16, 1968.

PREVIOUS studies (Pound and Withers, 1963; Pound, 1963, 1966) have shown that a single treatment of the skin of mice by scarification or chemical means, a short interval before a standard tumour-initiating treatment with urethane, augmented the yield of skin tumours. The augmenting effect was confined to the area affected by the preliminary treatment. The view advanced was that proliferating cells are more susceptible to the tumour-initiating action of urethane, since, when the preliminary treatment was made with croton oil, the number of tumours produced could be related to the cellular proliferation that occurred in the skin. In particular, it correlated with the number of cells replicating DNA at the time of injection of the urethane (Pound, 1966, 1968). The simplest hypothesis to explain these findings is that urethane acts during the replication of DNA. This may also be the case with the carcinogenic hydrocarbons (Pound, 1968).

This paper records the results of experiments to test the effect of preliminary irritation on the number of tumours produced by 3 carcinogenic hydrocarbons in single dosage without the use of a promoting treatment.

### MATERIALS AND METHODS

### *Mice*

Random bred male mice of the strain " Hall " (Pound, 1962) from the Department of Pathology at the Royal Brisbane Hospital were used. The animals were about 7 weeks of age and weighed 24–26 g. at the beginning of the experiments. They were housed in stainless steel compartments, each holding 10 mice, with a bed of coarse sawdust that was changed weekly, and were fed the diet previously used (Pound and Withers, 1963) supplied, with water, in excess of their needs. The animal house was air-conditioned at 22° C.

### *Chemicals*

Acetic acid, Osta Chemical Company, analytical reagent. Acetone, By-Products and Chemicals Pty. Ltd., analytical reagent. Croton oil, Stafford, Allen & Sons, London. 7,12-dimethylbenz(*a*)anthracene (DMBA); benzo(*a*)pyrene

(BP); and dibenz(*a,h*)anthracene (DBA) were obtained from L. Light & Co., London, and were used without further purification.

### EXPERIMENTAL

The hair of the skin of the back of the mice was clipped with electric clippers immediately before applications to the skin, care being taken to avoid injury since, in the light of previous work (Pound and Withers, 1963), this alone might influence the tumour yields. It was also clipped before counting tumours when necessary.

The hydrocarbons were applied to the whole area of the skin of the back by painting with approximately 0·25 ml. of solutions of the compounds in acetone: 0·6 %, 0·3 %, 0·15 % or 0·04 % in the cases of BP and DMBA, and 0·3 %, 0·15 % or 0·04 % in the case of DBA when the upper limit was defined by the solubility of the material. The area of skin covered was about 2·5 × 4·0 cm., that is about 10 sq. cm. The corresponding skin doses were therefore approximately 150, 75, 37, or 9 $\mu$g. per sq. cm. for BP and DMBA, and 75, 37, or 9 $\mu$g. per sq. cm. for DBA.

### *Experiments I, II and III*

One thousand and eighty mice were distributed at random into compartments of 10. The compartments were arranged at random into 3 lots of 12 groups of 30 mice, i.e. 1 lot for each of the 3 hydrocarbons: Experiment I, DBA; Experiment II, BP; and Experiment III, DMBA.

For each experiment the mice were divided into 4 divisions, each comprising 3 groups of 30 mice. The mice in each division were given a preliminary application of 0·25 ml. of a 25 % solution of acetic acid in acetone on the right side of the skin of the back, at 0, 24, 72 hours, or 9 days respectively before a single application of the carcinogenic hydrocarbon. The mice in the 3 groups of the 4 divisions were given an application of hydrocarbon to both sides of the skin of the back, 150, 37 or 9 $\mu$g. per sq. cm. for BP and DMBA and 75, 37 or 9 $\mu$g. per sq. cm. for DBA, respectively.

This gives a factorial arrangement in which the tumour yields on the 2 sides—treated and untreated with acetic acid—may be compared at 3 dose levels of the carcinogen and at 4 intervals of 0, 24, 72 hours and 9 days between the preliminary application of acetic acid and the application of the carcinogen. An interval of 0 hours between the 2 treatments means that the acetic acid solution was applied 15 minutes before the hydrocarbon, this being the time required for evaporation of the acetone solvent.

### *Experiment IV*

Six hundred mice were divided at random into 3 lots of 200 and each lot divided into 5 groups of 40 mice.

The mice of 4 groups in each lot were given a preliminary application of 0·5 ml. of a 0·5 % solution of croton oil in acetone to the whole area of the skin of the back at 0, 24, 72 hours and 9 days respectively, and the fifth group received no preliminary application, before the application of a carcinogenic hydrocarbon to the whole area of the skin of the back. The dose used was 75 $\mu$g. per sq. cm.

for all 3 hydrocarbons. One lot was treated with BP, the second lot with DMBA and the third lot with DBA.

*Experiment V*

Two control groups of 100 mice were painted once, one group with a 30% solution of acetic acid in acetone, the other group with 0·5% solution of croton oil in acetone. No carbinogenic hydrocarbons were administered.

The mice were examined at intervals for the presence of tumours in the treated area. A lesion was counted as a papilloma when it had reached a size of 1 mm. or more and persisted for 4 weeks or longer. A tumour was classified as malignant when it had grown progressively and had invaded the panniculus carnosus. Four sarcomata of the dermis developed in mice treated with the highest doses of DMBA but these are not included in the results. Malignant and doubtfully malignant growths were examined histologically but sections of clearly benign growths were not made. The number of tumours and their distribution on the skin of the back were recorded at fortnightly intervals, note being made of lesions that had regressed.

### RESULTS

The application of croton oil or acetic acid in the amounts used leads promptly to acute inflammation in the skin. Epithelial hyperplasia soon begins and leads to keratin scaling from about the 4th or 5th day (Pound, 1968). In the control Experiment V, no tumours appeared during 40 weeks observation of the mice that had a single application of acetic acid (75 survivors) or croton oil (82 survivors). The natural incidence of skin papillomata in this strain of mouse at 12 months of age is less than one in a thousand over the whole area of the body (personal observation).

The smallest doses (9 $\mu$g. per sq. cm.) of the carcinogenic hydrocarbons did not produce any clinically obvious changes in the skin and did not alter the changes visible to the naked eye in areas previously treated with croton oil or acetic acid. The intermediate doses produced some epithelial scaling after about 4 or 5 days and was least with DBA and BP. It was most noticeable with DMBA when it was accompanied by some serous oozing in a few mice, mainly in areas that had been treated with acetic acid or croton oil. The largest doses of DBA and BP produced obvious keratin scaling but visible ulceration did not occur. The largest dose of DMBA (150 $\mu$g. per sq. cm.) produced severe changes in the skin submerging the effects of the preliminary treatments. There were often large areas of serous exudation which were slow in returning to normal, hair regrowth was delayed or in some cases did not occur, and the changes were accompanied by a constitutional disturbance.

The mice treated with the highest dose of DMBA did not gain as much in weight as those in the other groups. At the end of 30 weeks the animals were not in very good condition and, as the death rate appeared to be increasing, the experiments with DMBA were terminated at this time. Three of the mice treated with the highest dose of DMBA developed sarcomas associated with the thoracic cage and 4 of them developed a total of 6 papillomata well outside the treated area (which are not counted in the results). The occurrence of these tumours must be ascribed to absorption of the DMBA, which also may account for the deterioration in condition of these animals. In all the other groups the mice remained in better condition and the death rates were not so great. These experiments were continued for 40 weeks.

In the mice of Experiment III treated with 150 $\mu g$. per sq. cm. of DMBA and a preliminary application of acetic acid at the same time and 1 day beforehand, a crop of small papillary growths occurred on the skin from the 8th week, a total of 7 on the side treated with acetic acid and 2 on the untreated side; these lesions regressed rapidly and do not appear in the results. This crop of transient lesions was not seen in Experiment IV in the mice given the preliminary application of croton oil before DMBA.

The time of appearance of each tumour counted, the tumours that regressed and those that became malignant, on each mouse, are set out in Tables I to IV. The mice that died with tumours are also shown.

The statistical analysis for Experiments I, II and III is based on the actual counts of tumours at each 10-week interval recorded in Table II; and for Experiment IV on the actual count at the termination of the experiment, Table IV.

*Experiments I, II and III*

Although the mice in these 3 experiments were randomized, the tumour yields were too small to allow the data to be treated as a whole and the experiments were considered individually.

*Experiment I—DBA.*—The number of tumours was too small to estimate the effects of the various factors. At 40 weeks the total of 12 tumours on the right side treated with acetic acid, combining the results of the 3 dose levels, is significantly greater than the total of 3 tumours on the left untreated side ($\chi_1{}^2 = 5 \cdot 4$, $P < 0 \cdot 05$).

*Experiment III—DMBA.*—Firstly, it was noted that the number of tumours on the right side is significantly greater than on the left side, after 10 weeks, 20 weeks and 30 weeks for dose 150 $\mu g$. per sq. cm. and after 20 weeks and 30 weeks for dose 37 $\mu g$. per sq. cm.

The tumour yields were treated as Poisson type counts, and analysis of variance performed after the square root transformation was applied.

The analysis showed that the differences between the right side and the left side, for the dose 150 $\mu g$. per sq. cm., were not related to the number of weeks of observation ($F_{2 \cdot 6} = 0 \cdot 77$) but depended on the length of the interval between treatments ($F_{3 \cdot 6} = 22 \cdot 51, P < 0 \cdot 01$). For dose 37 $\mu g$. per sq. cm. the difference between right and left sides again depended on the interval between treatments ($F_{3 \cdot 3} = 92 \cdot 49$, $P < 0 \cdot 01$), but was not related to the period of observation ($F_{1 \cdot 3} = 7 \cdot 67, P > 0 \cdot 5$).

*Experiment II—BP.*—A similar analysis was made to that for DMBA using the 20-, 30- and 40-week data for dose level 150 $\mu g$. per sq. cm. but only the 30- and 40-week data for dose 37 $\mu g$. per sq. cm. At 40 weeks the total yield of 22 tumours, combining the results of all dose levels, on the right side is significantly greater than the 9 tumours on the left side. Analysis of variance of the right *versus* left side differences at the 150 $\mu g$. per sq. cm. dose level gave no significant effect for weeks of observation ($F_{2 \cdot 6} = 0 \cdot 87$) but a significant effect for interval between treatments ($F_{3 \cdot 6} = 17 \cdot 37$, $P < 0 \cdot 01$). For the 37 $\mu g$. per sq. cm. dose level neither effect is significant (for intervals between treatments $F_{3 \cdot 3} = 5 \cdot 20$, $P > 0 \cdot 1$), presumably because of the smallness of the yields.

*Experiment IV*

To consider the results of the individual carcinogens, in the mice treated with DMBA there are significant differences between the tumour yields ($\chi_4^2 = 14 \cdot 236$, $P < 0 \cdot 01$). The control group, given no preliminary treatment, differs significantly from the pretreated groups ($\chi_1^2 = 5 \cdot 074$, $P < 0 \cdot 05$) and there are significant differences between the pretreated groups ($\chi_3^2 = 9 \cdot 162$, $P < 0 \cdot 05$).

TABLE I.—*Influence of a Preliminary Application of Acetic Acid at Intervals before Application of Hydrocarbon on Distribution and Time of Appearance of Tumours*

| Carcinogen | Dose. µg. per sq. cm. | Interval between treatments | Survivors at 20 weeks | Distribution and time of appearance of tumours on each mouse (Left side/Right side) |
|---|---|---|---|---|
| DBA | 75 | 0 | 28 | 34/0; 0/*24*; 0/*22*; 0/28, (22); 0/30 |
| | | 1 | 26 | 36/0; 0/26; 0/32 |
| | | 3 | 27 | 32/0; 0/32 |
| | | 9 | 25 | 0/(20); 0/34 |
| | 37 | 0 | 27 | 0/*22* |
| | | 1 | 23 | (22)/0; 0/32 |
| | | 3 | 23 | No tumours. |
| | | 9 | 28 | 0/24 |
| | 9 | 0 | 30 | 0/28 |
| | | 1 | 29 | No tumours. |
| | | 3 | 25 | 0/(24); (28)/0 |
| | | 9 | 30 | No tumours. |
| BP | 150 | 0 | 26 | 0/36; 34/28, *16*; 0/16, *18*; 24/*16*, 24; 0/32 |
| | | 1 | 27 | 28/0; 34/*24*; 0/18, 30; 0/26; 0/36 |
| | | 3 | 27 | (26), 28/*16*, *18*; 0/30 |
| | | 9 | 27 | 28/0; 0/(30) |
| | 37 | 0 | 23 | 28/30; 0/28, 34 |
| | | 1 | 28 | 24/0; 0/26, 24 |
| | | 3 | 30 | No tumours. |
| | | 9 | 28 | 36/0 |
| | 9 | 0 | 25 | No tumours. |
| | | 1 | 27 | 0/26 |
| | | 3 | 27 | No tumours. |
| | | 9 | 27 | No tumours. |
| DMBA | 150 | 0 | 23* | 20/(12), 22, 26; 0/22; 0/*12*; 0/*12*; 0/*12*, *16*, (12); [0/18]; 12/*12*; 0/*16*, 16, 18, 24; 0/(14); 0/22; 18/(22); 0/20, (18); 0/*14*, *16*, 24; [0/*16*, *16*]; [14/16, 18] |
| | | 1 | 22 | 0/22; 0/10, 14; (10)/(12), *12*, 14; 24/0; 24/(12); 0/10; 20/0; 22/(10); 0/(12), *16*; 0/22; [0/*16*, 22] |
| | | 3 | 22 | 22/0; 0/*16*, 19; 0/22, (12), (14); *12*, (14)/14; 0/22; 0/*22*, (10); 24/(22); 0/(18); 0/*16* |
| | | 9 | 28 | (10)/0; 16/0; 14/10; 22/(10); [0/18]; *12*/0; (24)/0; 0/*12* |
| | 37 | 0 | 25 | 0/14; 22/*12*, 18; 0/18; 0/16; 0/(16); 0/*12*; 22/0; 0/20, 22 |
| | | 1 | 28 | 0/16; 0/18, 18, 18, 24; 0/24 |
| | | 3 | 24 | (16)/0 |
| | | 9 | 26 | 0/24; 24/26; 22/0; 24/22, (24) |
| | 9 | 0 | 28 | 0/22; 0/(18) |
| | | 1 | 28 | 0/*14*; 0/24 |
| | | 3 | 28 | No tumours. |
| | | 9 | 28 | (12)/0; 22/(16) |

Figures in parentheses are times of appearance of tumours that regressed.
Figures in italics are times of appearance of tumours that became malignant.
Figures in square brackets refer to mice that died with tumours.
* Tumours that appeared at ten weeks and regressed before twenty weeks are not shown.

TABLE II.—*The Influence of Preliminary Treatment with Acetic Acid on Number of Tumours Produced by Hydrocarbons*

| Carcinogen | Interval between treatments | DBA 75 μg. per sq. cm. BP and DMBA 150 μg. per sq. cm. — Surviving mice: Number of mice | Number of tumours: Left side | Number of tumours: Right side | 37 μg. per sq. cm. — Surviving mice: Number of mice | Number of tumours: Left side | Number of tumours: Right side | 9 μg. per sq. cm. — Surviving mice: Number of mice | Number of tumours: Left side | Number of tumours: Right side |
|---|---|---|---|---|---|---|---|---|---|---|
| DBA (40 weeks) | 0 | 25 | 1 | 4 | 24 | 0 | 1 | 25 | 0 | 1 |
| | 1 | 24 | 1 | 2 | 22 | 0 | 1 | 28 | 0 | 0 |
| | 3 | 25 | 1 | 1 | 23 | 0 | 0 | 21 | 0 | 0 |
| | 9 | 22 | 0 | 1 | 24 | 0 | 1 | 27 | 0 | 0 |
| DBA (30 weeks) | 0 | 28 | 0 | 3 | 26 | 0 | 1 | 26 | 0 | 1 |
| | 1 | 26 | 0 | 1 | 22 | 0 | 0 | 28 | 0 | 0 |
| | 3 | 26 | 0 | 0 | 23 | 0 | 0 | 23 | 1 | 1 |
| | 9 | 23 | 0 | 1* | 26 | 0 | 1 | 28 | 0 | 0 |
| BP (40 weeks) | 0 | 21 | 2 | 8 | 23 | 1 | 3 | 24 | 0 | 0 |
| | 1 | 23 | 2 | 5 | 26 | 1 | 2 | 26 | 0 | 1 |
| | 3 | 24 | 1 | 3 | 25 | 0 | 0 | 24 | 0 | 0 |
| | 9 | 25 | 1 | 0 | 27 | 1 | 0 | 23 | 0 | 0 |
| BP (30 weeks) | 0 | 21 | 1 | 6 | 23 | 1 | 2 | 24 | 0 | 0 |
| | 1 | 23 | 1 | 4 | 28 | 1 | 2 | 27 | 0 | 1 |
| | 3 | 25 | 2 | 3 | 30 | 0 | 0 | 27 | 0 | 0 |
| | 9 | 25 | 1 | 1 | 27 | 0 | 0 | 27 | 0 | 0 |
| BP (20 weeks) | 0 | 26 | 0 | 4 | 23 | 0 | 0 | 25 | 0 | 0 |
| | 1 | 27 | 0 | 1 | 28 | 0 | 0 | 27 | 0 | 0 |
| | 3 | 27 | 0 | 2 | 30 | 0 | 0 | 27 | 0 | 0 |
| | 9 | 27 | 0 | 0 | 28 | 0 | 0 | 27 | 0 | 0 |
| DMBA (30 weeks) | 0 | 19 | 3 | 17 | 24 | 2 | 8 | 25 | 0 | 1 |
| | 1 | 17 | 4 | 8 | 23 | 1 | 6 | 24 | 0 | 2 |
| | 3 | 16 | 3 | 7 | 24 | 0 | 0 | 28 | 0 | 0 |
| | 9 | 22 | 4 | 2 | 26 | 3 | 3 | 26 | 1 | 0 |
| DMBA (20 weeks) | 0 | 23 | 4 | 16 | 25 | 1 | 6 | 28 | 0 | 1 |
| | 1 | 22 | 1 | 10 | 28 | 0 | 4 | 28 | 0 | 1 |
| | 3 | 22 | 1 | 4 | 24 | 1 | 0 | 28 | 0 | 0 |
| | 9 | 28 | 4 | 3 | 26 | 0 | 0 | 28 | 1 | 1 |
| DMBA (10 weeks) | 0 | 23 | 2 | 22 | 27 | 0 | 0 | 30 | 0 | 0 |
| | 1 | 26 | 2 | 10 | 29 | 0 | 0 | 29 | 0 | 0 |
| | 3 | 28 | 4 | 9 | 26 | 0 | 0 | 29 | 0 | 0 |
| | 9 | 29 | 1 | 2 | 29 | 0 | 0 | 30 | 0 | 0 |

30 mice in each group at beginning of experiment. * Present at 20 weeks.

TABLE III.—*Influence of a Preliminary Application of Croton Oil at Various Intervals before Application of Hydrocarbons on the Yield of Tumours and Times of Appearance of Tumours*

| Carcinogen | Dose. µg. per sq. cm. | Interval between treat-ments | Survivors at 20 weeks | Distribution and time of appearance of tumours on each mouse (Right side/Left side) |
|---|---|---|---|---|
| DBA (40 weeks) | 75 | 0 | 33 | 34/0; 28/*20*, (32); 34/0; 28/0; (32)/0; [0/20] |
| | | 1 | 34 | 34/0; 28/36, (34); 0/22 |
| | | 3 | 32 | 36/0; 22/0; 0/28 |
| | | 9 | 37 | No tumours. |
| | | A | 36 | 36/0; (24)/0; 0/32 |
| BP (40 weeks) | 75 | 0 | 34 | 24/0; 22/18; 18, 22/22, 32, (24); 0/36; [(22)/*16*, 22] |
| | | 1 | 34 | 36/0; 24/18; 32/*18*, 24; 28/24, 28; (24)/36; 0/24, 34 |
| | | 3 | 34 | (20)/0; *18*, 32/28; 26/24, 32; 0/24 |
| | | 9 | 35 | 22, 28, 36/0; 0/30; 0/32 |
| | | A | 39 | 28/0; 32/36, (22) |
| DMBA (30 weeks) | 75 | 0 | 29 | *12*, 18/0; 18/(22), 24; 24/0; 20/*14*, *18*, (18); (16), 22/16; 0/14; [0/*12*, 16]; [*10*, 18, 20/0] |
| | | 1 | 33 | *12*, 16/18; *16*, *18*, (18), 24/12, 22; 16/20; 0/*18*, 22; 24/0; 16/*14*; 16, 24/20, 22; 18/0; 22, 26/0; [24/0] |
| | | 3 | 28 | 16, (18), (18)/20; 20, 22/0; 12/18, (22); 26/18, 24; 0/18, 22; 0/12, (20); (20)/24 |
| | | 9 | 30 | 12/*10*; 22/0; 20/18, (18), (24), 24; (16)/16, 24 |
| | | A | 34 | 12/0; 20/*14*, (18); 16/22; 24/0; (24)/(24) |

Figures in parentheses are times of appearance of tumours that regressed.
Figures in italics are times of appearance of tumours that became malignant.
Figures in square brackets refer to mice that died with tumours.
Forty mice in each group at beginning of experiment.
Groups A had no preliminary treatment with croton oil.

TABLE IV.—*The Influence of Preliminary Treatment with Croton Oil on Number of Tumours Produced by Hydrocarbons*

| | Interval between treatments | Surviving mice | | |
|---|---|---|---|---|
| | | Number of mice | Mice with tumours | Number of tumours |
| DBA, 75 µg./sq. cm. (40 weeks) | 0 | 29 | 4 | 5 |
| | 1 | 29 | 3 | 4 |
| | 3 | 28 | 3 | 3 |
| | 9 | 31 | 0 | 0 |
| | A | 32 | 2 | 2 |
| BP, 75 µg./sq. cm. (40 weeks) | 0 | 31 | 4 | 8 |
| | 1 | 29 | 6 | 12 |
| | 3 | 27 | 3 | 5 |
| | 9 | 30 | 3 | 5 |
| | A | 34 | 2 | 3 |
| DMBA, 75 µg./sq. cm. (30 weeks) | 0 | 25 | 6 | 11 |
| | 1 | 27 | 9 | 22 |
| | 3 | 26 | 7 | 13 |
| | 9 | 28 | 4 | 8 |
| | A | 30 | 4 | 6 |

Groups A had *no* preliminary treatment. Forty mice in each group at beginning of experiment.

In the mice treated with BP no very significant differences appear (overall $\chi_4^2 = 8 \cdot 428$, $P > 0 \cdot 05$; difference between control and pretreated groups $\chi_1^2 = 3 \cdot 409$, $P > 0 \cdot 05$; differences between pretreated groups $\chi_3^2 = 5 \cdot 019$, $P > 0 \cdot 10$). Similarly no significant differences appear in the mice treated with DBA.

For an analysis of variance, if it is assumed that the difference due to the intervals between treatments can be estimated independently of the carcinogen used, the data can be treated as a $3 \times 5$ factorial (3 carcinogens *versus* 4 pre-treatments and a control group). Tumour yields were treated as Poisson type counts.

Significant differences appear between the 3 carcinogens ($F_{2 \cdot 8} = 21 \cdot 43$, $P < 0 \cdot 001$). There are also significant differences between the control groups A and the pretreatment groups ($F_{1 \cdot 8} = 7 \cdot 414$, $P < 0 \cdot 05$). In addition, significant differences exist between the 4 pretreatment groups ($F_{3 \cdot 8} = 6 \cdot 489$, $P < 0 \cdot 05$). Comparing the pretreated groups with the controls the differences at the 0 interval and the 1 day interval are significant, $P < 0 \cdot 05$, and $P < 0 \cdot 01$ respectively. The difference between the controls and the 3 day interval is of doubtful significance and the remaining difference not significant at all.

In summary, there is clear evidence that, when one side of the skin of the back of mice is treated with acetic acid before application of any of the 3 hydrocarbons to the whole area of the skin of the back, the number of tumours produced is greater on the pretreated side. In the case of DMBA at the 150 $\mu$g. per sq. cm. and 37 $\mu$g. per sq. cm. dose levels and in the case of BP at the 150 $\mu$g. per sq. cm. dose level the increase varied with the interval between the 2 treatments and was independent of the period of observation. The tumour yields were increased when there was no significant interval between the 2 treatments and at an interval of 24 hours. A similar trend appears at the lower dose levels of DMBA and BP and also when DBA was the carcinogen although the tumour yields were too small for statistical treatment.

Similarly when mice were given a preliminary treatment with croton oil before the application of the carcinogens the tumour yield was increased when the interval between treatments was zero and 24 hours. A doubtful effect was found at an interval of 3 days. The variation with the interval between treatments is significant only with DMBA, but a similar trend is seen in the results with DBA and BP.

From inspection of Tables I and III it is apparent that these trends are maintained in mice that died with tumours. Also, the number of tumours that regressed was greater on skin that had the preliminary treatment.

It is to be noted that the tumour yields in areas of skin that did not have the preliminary treatment with acetic acid (in Experiments I, II, and III), appear to be similar, allowing for variation in dosage levels, to the tumour yields in mice that had no pretreatment with croton oil in Experiment IV. Similarly, in areas of skin subjected to preliminary treatment with acetic acid in Experiments I, II and III the increase in tumour yields is of similar order to the increase in the tumour yields in similar areas of mice that had a preliminary treatment with croton oil, again allowing for the variation in dosage levels.

It will be observed from Tables I and III that tumours appear earlier on the average with the more potent carcinogens. The tumour yields are not such as to treat this statistically.

Lastly, it is apparent from Tables I and III that malignant tumours appear to be more frequent in areas given the preliminary treatment on the one hand and with the more potent carcinogens on the other. However, the ratio of malignant to benign tumours does not vary significantly between the different areas. The frequency of malignant tumours therefore appears to be related to the number of tumours in any particular area, and perhaps also to the length of time tumours have been under observation.

### DISCUSSION

The production of skin tumours in mice by a single application of a carcinogenic hydrocarbon has been reported, for example, using 3 : 4-benzopyrene (Bielschowsky and Bullough, 1949), 20-methylcholanthrene (Mider and Morton, 1939; Cramer and Stowell, 1943) and 9 : 10-dimethyl-1 : 2-benzanthracene (Law, 1941; Andreasen and Engelbreth-Holm, 1953; Borum, 1954; Terracini, Shubik and Porta, 1960). The tumours appeared in small numbers after a long latent period; most have been benign but some were malignant. Allowing for the differing strains of mice used by different workers and for the uncertainties of dosage per unit area of skin, DMBA appears to be the most active as judged by the numbers of tumours produced.

The number of tumours to be expected in the experiments reported in this paper is therefore likely to be small, as was found, although a quantitative comparison of the results with those of other authors is invalid because of the differing strains of mice, uncertainty in the dosage per unit area used by other authors, the particular sample of DMBA, DBA and BP used and possibly other factors. Dose-response data have been reported with DMBA (Terracini *et al.*, 1960) and the present results follow a similar pattern. Nonetheless, it is clear that DMBA produced more tumours than DBA or BP, and that BP was more active than DBA, at least with the samples used.

A preliminary application of acetic acid or croton oil at the same time as, or a short interval before, the application of any of the 3 hydrocarbons increased the number of tumours produced. The relative increase when the treated side of mice that had the preliminary treatment of acetic acid on one side of the skin of the back was compared with the untreated side was similar to the relative increase when mice that had a preliminary treatment with croton oil to the whole of the back were compared with mice that had no preliminary treatment. The augmenting effect is therefore localized to the area affected by the preliminary treatment and not the result of general metabolic changes; although formal demonstration of this would depend on the results of similar experiments in which the manner of the preliminary treatments were reversed. However, since the tumour yields in untreated areas of skin in the 2 experiments were similar, these experiments would appear to be redundant. The amounts of acetic acid or croton oil applied were the highest that could be used without producing clinical ulceration of the skin and led to similar degrees of epithelial scaling as judged by the naked eye.

Croton oil is a potent promoting agent for the production of skin tumours in mice subjected to the action of an initiating agent such as urethane (Salaman and Roe, 1953) or the carcinogenic hydrocarbons (Mottram, 1944*a*; Berenblum and Shubik, 1947); and on repeated application alone appears to have a minor carcinogenic effect (Roe, 1956; Boutwell, Bosch and Rusch, 1957). However, acetic acid has no promoting activity when carcinogenic hydrocarbons are employed as initiating agents and does not itself lead to the production of tumours (Gwynn and Salaman, 1953). The tumour augmenting effect is therefore, as in the case of the similar augmenting effect of preliminary irritation on urethane carcinogenesis (Pound and Withers, 1963; Pound, 1966), not related to these properties but is probably related to the common property of producing inflammation and cell proliferation in the skin.

However, in the case of experiments in which hydrocarbons are applied to the skin, other factors merit consideration. Thus, the number of tumours produced after a single application of DMBA appears to be related to the stage of the hair cycle at the time, more tumours being produced if this is in the resting phase (Andreasen and Engelbreth-Holm, 1953; Borum, 1954). From study of sections of mice of the same age and weight, the hair cycle of about one-third the mice used in the present experiments is in the late catagen phase and of the remainder in the resting phase. The possibility that the results can be due to a variation from this source is therefore unlikely and can be excluded since treated sides were compared with the untreated sides of the same animals in which the cycle would be in the same phase on either side. However, it has been shown that the hair cycle effect is explained mainly by retention of the hydrocarbon in the resting hair follicles (Berenblum, Haran-Ghera and Trainin, 1958).

After the application of croton oil in the amount used, vascular dilatation and oedema develop rapidly and increase to about the 12th hour, after which the changes regress slowly to approach normal after about 24 hours. These changes are accompanied by a leucocytic infiltration which, however, persists for longer than 24 hours. The number of nuclei of the epidermis labelled in radio-autographs of sections of the skin, when the mice were injected with 10 $\mu$c tritiated thymidine 30 minutes before being killed, increased very rapidly from the 9th hour to a maximum at the 18th hour and then receded to normal at about the 9th day, whereas mitotic counts in the epidermis increased slowly from the 15th hour to a maximum at about 36 hours and then receded slowly to normal at about the 9th day (Pound, 1968). A similar pattern of these events was reported by Iversen and Evensen (1962). Forty-eight hours after the application of croton oil the epidermis is increased in thickness and soon begins to differentiate a layer of keratin. The epidermal changes extend into the superficial part of the hair follicles. Although no study has been made of the changes following an application of acetic acid, the changes visible to the naked eye are similar and the microscopic events are likely to be substantially the same.

It seems possible that the proliferating epidermis, on the surface and in the superficial part of the hair follicles, might have an increased capacity to retain hydrocarbons applied to the skin but if this factor contributed significantly to the results, the tumour yield would be expected to be augmented when the hydrocarbon was applied on the 3rd and 9th days after the preliminary application. This was not the case.

In previous studies concerning the augmenting effect of irritants on tumour initiation by urethane (Pound, 1968) it has been shown that the increased tumour yield correlates with cellular events in the skin, in particular with the number of cells replicating DNA, at the time of injection of the urethane. This suggests that this chemical exerts its tumour initiating action during this phase.

It seems possible that the carcinogenic hydrocarbons also act during a similar period of the cell cycle. The demonstration of this in experiments in which the hydrocarbon is applied to the skin is likely to be clouded by the facts that, on the one hand, these compounds persist in the tissue for a considerable time, and, on the other hand, they themselves lead to epithelial hyperplasia in the skin (Orr, 1938). The sharp increase in tumour yields found when urethane was injected as an initiating agent at various times after a preliminary irritating treatment of the skin could not be expected. This indeed was the reason for selection of the particular intervals between the preliminary treatment and the application of the hydrocarbon used in the present experiments.

Many years ago Mottram (1944*b*) reported that the number of tumours produced by benzopyrene (used as an initiating agent followed by repeated applications of croton oil which he referred to as a developing factor, but which is now referred to as a promoting agent) was increased if the skin was given a preliminary treatment with croton oil. He showed that the preliminary treatment resulted in a great increase in the mitotic counts in the skin. Similarly, he found that a preliminary treatment with cantharidin under conditions that depressed the mitotic counts decreased the tumour yields, and under conditions that increased the mitotic counts cantharidin increased the tumour yields. Mottram (1945) also found that more tumours were produced if the benzopyrene was applied at midnight rather than at midday which he ascribed to higher mitotic rates at this time, a view that has not been supported by subsequent results (Bielschowsky and Bullough, 1949). He concluded that " the genesis of tumours represents action on cell division ". In spite of the revolutionary nature of this concept, for various reasons experiments that would confirm or refute this thesis have only been performed recently.

Frei and Ritchie (1964) have confirmed that DMBA, followed by promoting treatment with croton oil, produces more tumours if applied at midnight than if applied at midday; and Shinozuka and Ritchie (1967) have reported that a preliminary application of croton oil 23 hours before the application of DMBA, as an initiating agent, augmented the yield of tumours. These results were interpreted as compatible with the theory that the carcinogens act on cells synthesizing DNA.

A similar result was reported (Pound, 1968) using DBA as an initiating agent but the tumour yield was increased at intervals of 0, 24 hours and 3 days, and not at 9 days, between a preliminary application of acetic acid and the carcinogen. Almost identical results have been obtained (Pound, to be published) when BP and DMBA were the initiating agents. While these results completely dissociate the augmenting effect of the preliminary treatment from promoting activity, they do not give the clear correlation with the pattern of DNA synthesis found with urethane. Nor should such a pattern be expected for reasons noted above. In further experiments (Pound, 1968) it was reported that partial hepatectomy before injection of urethane or oral administration of DMBA increased the number of tumours produced in the liver, an effect that may be related to the active regeneration of the liver during the presence of the carcinogen.

It seems possible therefore that tissues induced to proliferate rapidly become more susceptible to the action of a carcinogen, as a general phenomenon. The evidence that possibly identifies the sensitive phase in cell life as the phase of replication of DNA comes mainly from the work with urethane (Pound, 1968).

### SUMMARY

Groups of mice were given a single application of acetic acid to one side of the skin of the back. Other groups were given an application of croton oil to the whole area of the skin of the back, a control group had no application of croton oil. At the same time as, 24 hours, 72 hours, or 9 days after the application of acetic acid or croton oil, the mice were given a single application of one of the carcinogenic hydrocarbons DMBA, BP or DBA, to the whole area of the skin of the back.

The number of tumours produced was greater in areas that had the preliminary treatment with acetic acid or croton oil at the same time or 24 hours before the carcinogen. There was a doubtful effect at an interval of 3 days and no effect at 9 days.

It is considered that proliferating epidermal cells are more susceptible to the action of the carcinogens perhaps during replication of DNA.

The author thanks Dr. H. Silverstone, Reader in Medical Statistics, for the statistical analysis. This work was partly supported by a grant from the Queensland Cancer Fund.

### REFERENCES

ANDREASEN, E., AND ENGELBRETH-HOLM, J.—(1953) *Acta path. microbiol. scand.*, **32**, 165.

BERENBLUM, I., HARAN-GHERA, NECHAMA, AND TRAININ, N.—(1958) *Br. J. Cancer*, **12**, 402.

BERENBLUM, I., AND SHUBIK, P.—(1947) *Br. J. Cancer*, **1**, 379.

BIELSCHOWSKY, F. AND BULLOUGH, W. S.—(1949) *Br. J. Cancer*, **3**, 282.

BORUM, K.—(1954) *Acta path. microbiol. scand.*, **34**, 542.

BOUTWELL, R. K., BOSCH, D. AND RUSCH, H. P.—(1957) *Cancer Res.*, **17**, 71.

CRAMER, W. AND STOWELL, R. E.—(1943) *Cancer Res.*, **3**, 36.

FREI, J. V. AND RITCHIE, A. C.—(1964) *J. natn. Cancer Inst.*, **32**, 1213.

GWYNN, R. H. AND SALAMAN, M. H.—(1953) *Br. J. Cancer*, **7**, 482.

IVERSEN, O. H. AND EVENSEN, A.—(1962) *Acta path. microbiol. scand.* Supp. **156**.

LAW, L. W.—(1941) *Am. J. Path.*, **17**, 827.

MIDER, G. B. AND MORTON, J. J.—(1939) *Am. J. Path.*, **15**, 299.

MOTTRAM, J. C.—(1944*a*) *J. Path. Bact.*, **56**, 181.—(1944*b*) *J. Path. Bact.*, **56**, 391.

MOTTRAM, J. C.—(1945) *J. Path. Bact.*, **57**, 265.

ORR, J. W.—(1938) *J. Path. Bact.*, **46**, 495.

POUND, A. W.—(1962) *Br. J. Cancer*, **16**, 246.

POUND, A. W.—(1963) *Aust. J. exp. Biol. med. Sci.*, **41**, 73.

POUND, A. W.—(1966) *Br. J. Cancer*, **20**, 385.

POUND, A. W.—(1968) Proceedings of the First New Zealand International Symposium on Cancer, University of Otago, Dunedin, November, 1966. *N.Z. med. J.* (Special Issue), **67**, 88.

POUND, A. W. AND WITHERS, H. R.—(1963) *Br. J. Cancer*, **17**, 460.

ROE, F. J. C.—(1956) *Br. J. Cancer*, **10**, 72.

SALAMAN, M. H. AND ROE, F. J. C.—(1953) *Br. J. Cancer*, **7**, 472.

SHINOZUKA, H. AND RITCHIE, A. C.—(1967) *Int. J. Cancer*, **2**, 77.

TERRACINI, B., SHUBIK, P. AND PORTA, G. D.—(1960) *Cancer Res.*, **20**, 1538.